# FORTIFYING GERIATRIC CARE AND RESEARCH: THE VHA ADVANCED FELLOWSHIP IN GERIATRICS

**DOI:** 10.1093/geroni/igae098.0900

**Published:** 2024-12-31

**Authors:** Katherine Hall, Odessa Addison, Judith Howe

**Affiliations:** Chair; Co-Chair; Discussant

## Abstract

Geriatric care often involves addressing complex medical, social, and behavioral needs that require input from multiple disciplines. A robust workforce with geriatric specialties is essential to ensure that older adults receive the comprehensive care and support necessary for optimal aging. Despite the growing demand, there is a significant shortage of providers working in geriatric care, exacerbating the strain on existing resources. One way to address this shortage may be through the Advanced Fellowship in Geriatrics (AFiG), a Department of Veterans Affairs (VA) program overseen by the Office of Academic Affiliations (OAA). The AFiG is a 2-year, postgraduate (post-residency, post-doctoral, post-masters) training program, offered to clinicians and non-clinician scientists, that prepares leaders in geriatric research, education, and clinical innovation. Fellows are hosted in Geriatric Research, Education, and Clinical Centers (GRECC) in 20 VA medical centers across the country, providing a rich combination of clinical rotations, didactic education, and research opportunities. Fellows gain expertise in identifying and addressing the unique healthcare needs of older adults, particularly within the veteran population. This fellowship provides participants with a wealth of advanced training topics including management of multiple chronic conditions, population health and health promotion, models of care, policy initiatives, and interdisciplinary collaboration. This symposium features presentations from 1) AFiG leadership, 2) GRECC leadership, and 3) facilitated discussion with an interprofessional cohort of current/former Fellows to share the breadth of clinical and research experiences in the AFiG.

